# A manifest nodoventricular accessory pathway with unusual electrophysiological manifestations

**DOI:** 10.1002/joa3.12759

**Published:** 2022-07-18

**Authors:** Jun Li, Qifang Liu, Long Yang

**Affiliations:** ^1^ Department of Cardiology Guizhou Provincial People's Hospital Guiyang China

**Keywords:** 12‐lead ECG, decremental conduction, Intracardiac electrograms during atrial extra‐stimuli, Nodoventricular pathway, preexcitation

## Abstract

A nodoventricular accessory pathway showing the degree of anterograde decremental conduction was more than the atrioventricular node.

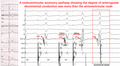

A 24‐year‐old male athlete with electrocardiographic preexcitation was referred to our center for electrophysiological evaluation. His electrocardiograms showed sinus rhythm with minimal preexcitation (Figure 1). During sinus rhythm, intracardiac electrograms revealed preexcitation with an A‐H interval 80 ms and H‐V interval of 20 ms. Ventricular overdrive pacing at a cycle length of 400 ms resulted in ventriculoatrial dissociation. Atrial extra‐stimuli (AES) were introduced during sinus rhythm with a cycle length of 500 ms, The coupling interval was reduced from 400 to 280 ms. This resulted in a gradual prolongation of the A‐H interval and the H‐V interval with a decrease in the degree of preexcitation and a change in the QRS complex morphology. These electrophysiologic findings ruled out fasciculoventricular pathway(FVP). Subsequently, spontaneous junctional beat was seen during electrophysiologic study, the H‐V interval was 28 ms, and the QRS complex morphology still remained minimal preexcitation, indicating the proximal insertion site of the accessory pathway was AV node and nodoventricular AP was diagnosed (Figure 2). Regarding this patient, neither rapid antegrade conduction nor inducible reentrant tachycardias were observed, and no ablation was attempted. Interestingly, the degree of anterograde decremental conduction of a nodoventricular AP was more than the atrioventricular node (AVN).

**FIGURE 1:**
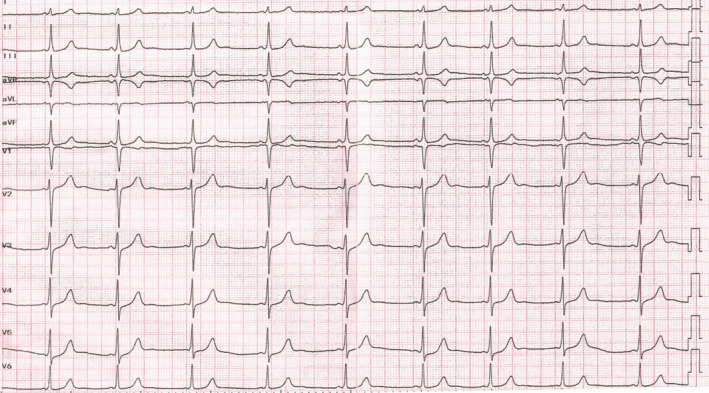
Twelve‐lead electrocardiographic recordings showing fixed and minimal preexcitation during sinus rhythm.

**FIGURE 2:**
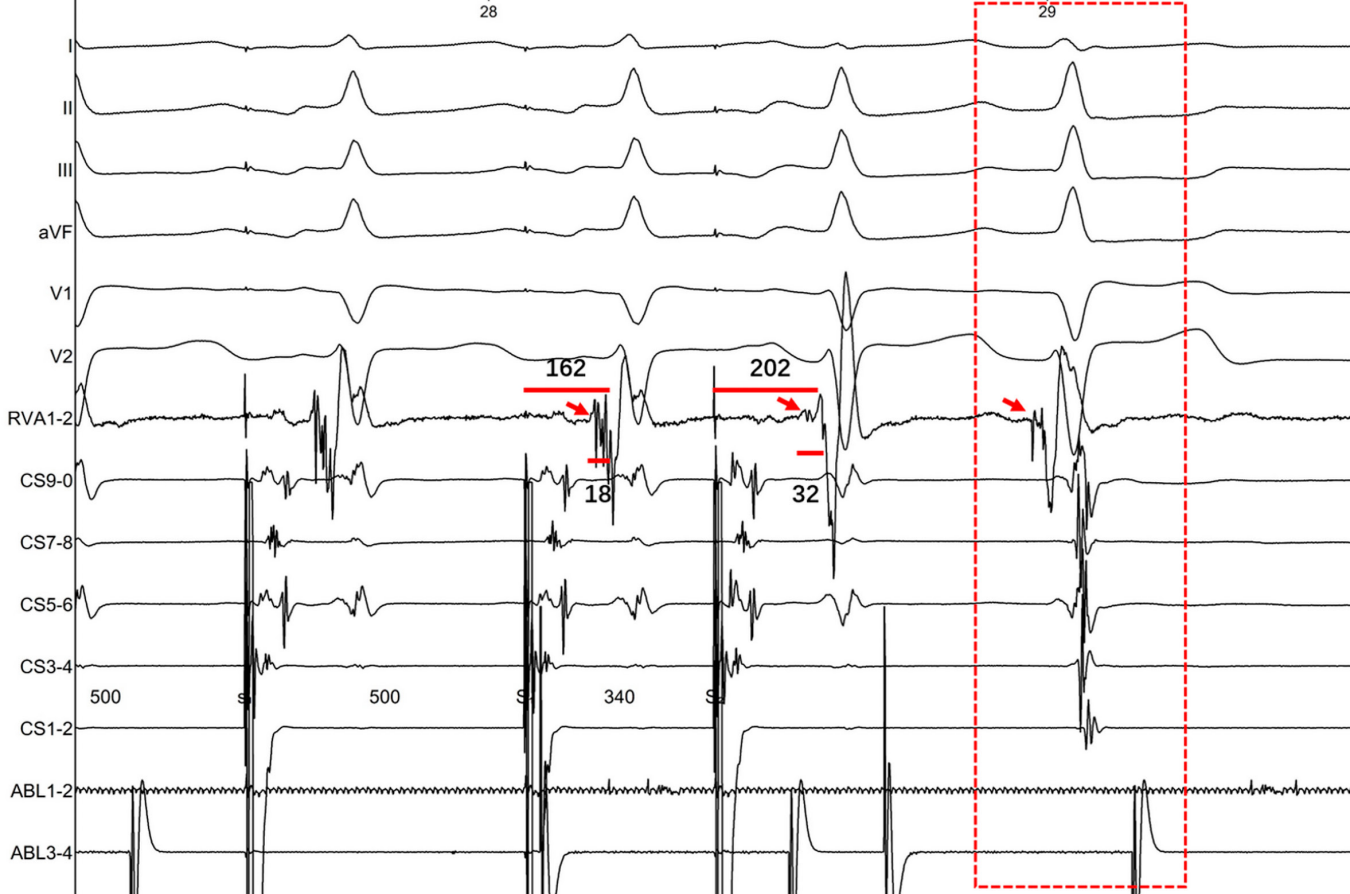
Intracardiac recordings during programmed atrial stimulation demonstrating decreased preexcitation with a prolongation of the H‐V interval. The last spontaneous junctional beat still showing a preexcitation morphology. Note that the QRS complex morphology change in the lead I and V1. His bundle electrograms are shown on the RV1‐2 recordings and red arrows indicate his potentials.

Nodoventricular pathways (NVP) are rare accessory pathways that connect the posterior extensions of the AVN to the crest of the ventricular septum.^1^ These pathways were anatomically described in 1941 by Mahaim and Winston. The diagnosis of manifest NVP is based on the following evidence: (1) upper insertion site is AVN, (2) lower insertion site is ventricular, and (3) proving accessory pathway presence with decremental conduction manifestations and excluding FVP.

In our case, the patient's 12‐lead ECG showed ventricular preexcitation, but without symptoms of arrhythmias. Programmed atrial pacing showed a significant AH prolongation associated with increased HV interval, excluding the diagnosis of FVP. Furthermore, the significant difference in the ventricular activation sequence and changing QRS morphology recorded during AES, favoring the diagnosis of an accessory pathway but an FVP (Figure 3). If the pathway was FVP, preexcitation and QRS morphology should remained fixed and H‐V remained constant when conducting over either fast or slow pathways. Catheter pressure over AVN region resulted in junctional ectopy that remained preexcitation, indicating the proximal insertion site of accessory pathway is AV node. These observations are consistent with an NVP.

**FIGURE 3:**
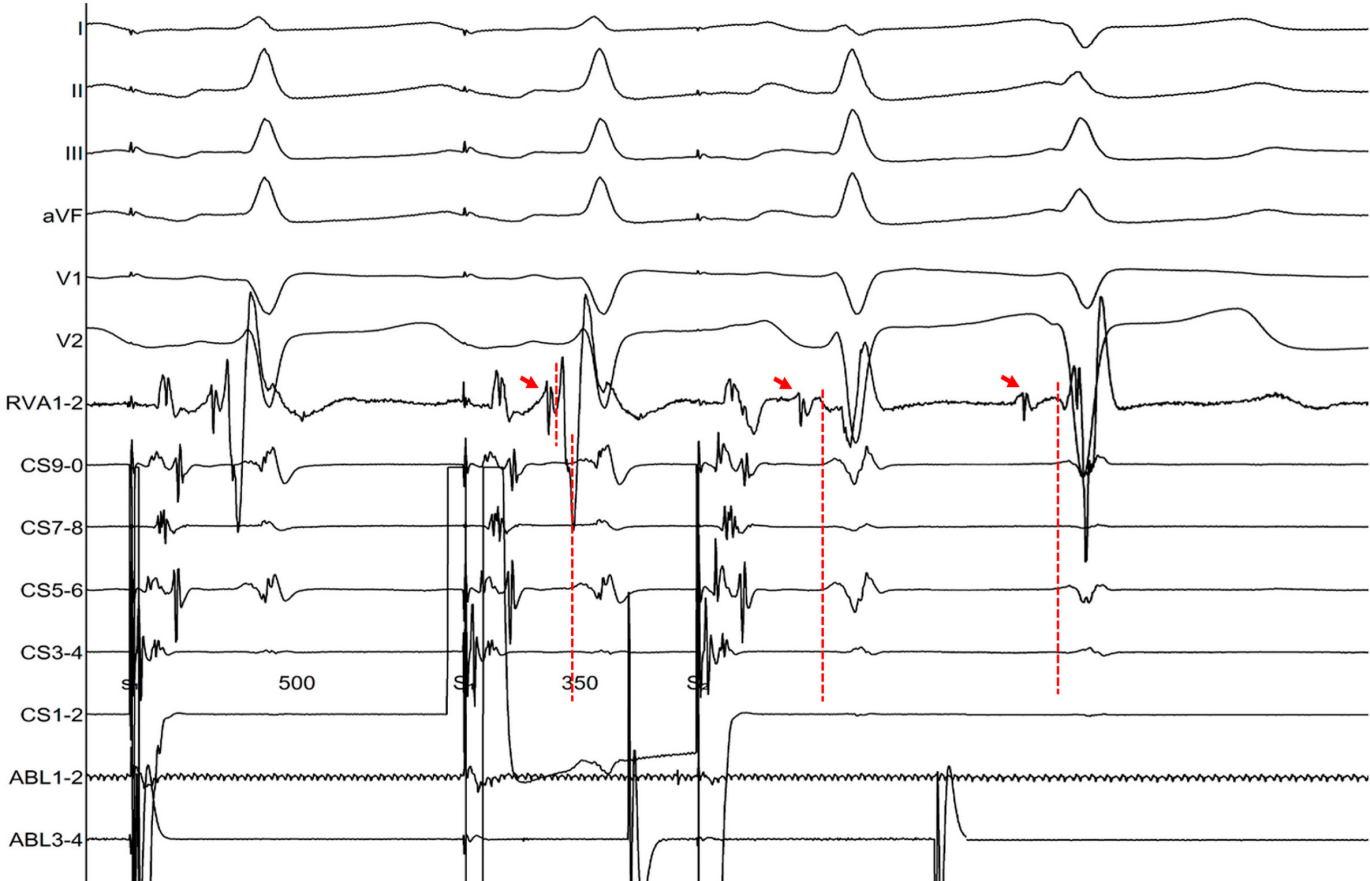
Intracardiac recordings during programmed atrial stimulation, demonstrating a longer HV interval and a significant difference in the ventricular activation sequence in his bundle electrograms and CS between the S1 and the S2. His bundle electrograms are shown on the RV1‐2 recordings and red arrows indicate his potentials.

In this case, on programmed atrial stimulation testing, there was decremental conduction over the pathway, resulting in a longer A–V and H‐V time as the A1–A2 coupling interval decreased. As far as we are aware, this case is unique since the nodoventricular fiber shows the degree of anterograde decremental conduction was more than the atrioventricular node. In contrast, most of the NVP bypass only a portion of the AV node with decremental conduction properties. Therefore, the preexcitation degree may increase and the H‐V interval may decrease even to negative values during AES.^2^ As far as our patient is concerned, the electrophysiologic features of anterograde decremental conduction may be due to the anisotropic conduction of the transitional area of the AV node and variability in the space construction of tissue.^3^ In addition, the differential expression of connexin isoforms in the nodal area also may be responsible for more degree decremental conduction of NVP than AVN.^4^

## FUNDING INFORMATION

This study was supported by the Clinical Research Center Project of the Department of Science and Technology of Guizhou Province.

## CONFLICT OF INTEREST

The authors declare that they have no conflict of interest.

## CONSENT FOR PUBLICATION

Patient consent for publication was obtained.
